# Subwavelength acoustic monopole source emission enhancement through dual gratings

**DOI:** 10.1038/s41598-019-48215-z

**Published:** 2019-08-12

**Authors:** Jun Mei, Ying Wu

**Affiliations:** 1 Department of Physics, South China University of Technology, Guangzhou, 510640 China; 2 King Abdullah University of Science and Technology (KAUST), Division of Computer, Electrical and Mathematical Science and Engineering (CEMSE), Thuwal, 23955-6900 Saudi Arabia

**Keywords:** Acoustics, Physics

## Abstract

Acoustic source emission rate is generally low at low frequencies. In this work, we propose a simple design of ‘LEGO’-type acoustic metamaterial that can significantly enhance the low frequency emission rate of an acoustic monopole source. Such enhancement is resulted from the coupling between resonances of a cavity and a dual grating comprised of two concentric layers of periodically distributed narrow slits. We develop an effective medium model to characterize the enhancement. Because of its simple structure, the metamaterial is easy to fabricate and thus facilitates the applications in various domains such as oil exploration.

## Introduction

Acoustic source emission rate is generally low at low frequencies because of its small size compared to the wavelength. The power radiated to the far field of an acoustic monopole source with size *D* is proportional to (*D*/*λ*)^2^, where *λ* is the wavelength, and the power decreases proportionally for higher multipoles^1^. Traditional means to enhance acoustic source emission rate usually include bulky structures, for example, a loudspeaker mouth adapted in a horn shape to improve the emission, and a woofer diaphragm mounted in a speaker cabinet to boost the low-frequency emission^2,3^. Very recently, a slab of acoustic metamaterial utilizing Fabry-Perot resonance was proposed to enhance the emission rate of a monopole source, but its planar geometry makes the emission enhancement not omnidirectional^4^. But such issue has been addressed by using a highly symmetric labyrinthine structure^5,6^. It can achieve low frequency enhancement of an acoustic source radiation because the elongated acoustic path effectively reduces the wave velocity along the radial direction. Despite of its good performance in the radiation enhancement, its complex structure adds complexity to the fabrication, which can be achieved only by 3D printing technology.

In this work, we propose a simple ‘LEGO’-type design of an acoustic metamaterial to enhance the low frequency source radiation. The enhancement relies on a new mechanism that couples the resonant modes of a cavity and periodically distributed narrow slits, which is different from the usual Fabry-Perot resonance and the elongated path effect. We find substantial emission enhancement in the low frequency regime, where the wavelength is about three times of the diameter of the metamaterial. Furthermore, our metamaterial has a very simple structure that is easy to fabricate, just like the LEGO toy bricks. Our design thus provides an alternative method for the realization of low frequency acoustic radiation enhancement, which will benefit potential applications in various fields such as oil exploration.

## Results

### Materials and Methods

**Figure 1:**
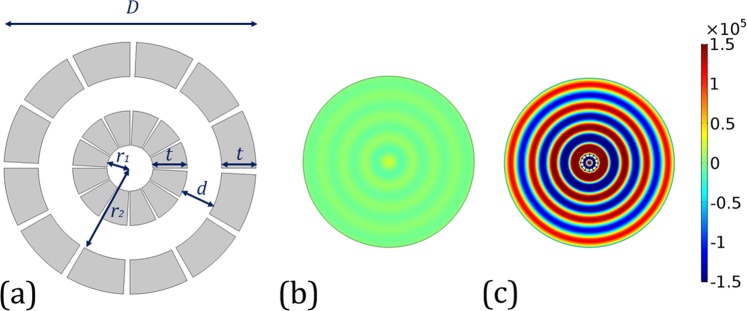
(**a**) Schematic of the two-layer enclosure. It consists of two concentric steel annuli with the same thickness, *t* = 3 mm, and the two annuli are separated by a *d* = 3 mm air spacer. The inner radii of these two annuli are *r*_1_ = 2 mm and *r*_2_ = 8 mm, respectively, and the diameter of the entire device is *D* = 22 mm. Both annuli have 12 cut-through slits, each of which occupies 3° and is distributed uniformly along the azimuthal direction. (**b**,**c**) The amplitude distribution of the pressure wave at 12300 Hz when a line monopole source is located at the center without (**b**) and with (**c**) the enclosure, respectively. It can be seen that the far-field radiation field is greatly enhanced after the enclosure is introduced.

The proposed design of the two-dimensional enclosure is schematically illustrated in Fig. 1(a). It consists of two layers of concentric steel annuli with the same thickness, *t* = 3 mm. The inner radii of these two annuli are *r*_1_ = 2 mm and *r*_2_ = 8 mm, respectively. Thus, the two annuli are separated by a *d* = 3 mm air spacer and the diameter of the entire enclosure is *D* = 22 mm. Both annuli have 12 cut-through slits, each of which occupies 3° and is distributed uniformly along the azimuthal direction. An acoustic line source is located at the center of the enclosure, which emits uniformly within the entire 2π radians range of the in-plane angle.

We use COMSOL Multiphysics, a commercial software based on the finite-element method, to conduct numerical simulations of wave propagation properties of the described system. Pressure acoustics module in the frequency domain is used in the simulation. The boundary between steel and air background is modulated as a sound hard boundary condition, which is justified by the huge acoustic impedance mismatch between air and steel. Monopole line source is set at the enclosure center, and a cylindrical wave radiation condition is specified at the outer boundary of the simulation domain to absorb the outgoing waves radiated by the line source.

### Enhanced source emission

Figure 1(b) shows the simulated far-field pressure field emitted by the acoustic line monopole source in free-space at frequency 12300 Hz. A typical cylindrical wave profile is observed. Here we use the term “far-field” whenever the near-field phenomena such as evanescent waves can be safely neglected. For practical calculations and simulations, a distance from the source that is at least several times of wavelength is considered to be in the far-field region. For comparison, the far-field pressure distributions are plotted in Fig. 1(c) when the same source is encompassed by the previously mentioned double-layer enclosure at the same frequency. Both Fig. 1(b,c) share the same color bar. It is obvious that Fig. 1(c) exhibits much larger magnitude of the far-field pressure at the same distance from the source, indicating the enclosure is able to enhance the acoustic source emission at this frequency.

**Figure 2:**
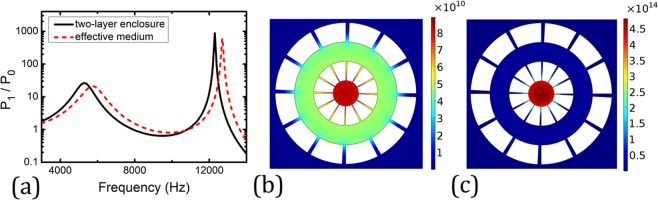
(**a**) The acoustic Purcell factor (black solid curve) by a line source located at the center of the enclosure shown in Fig. 1(a). Two peaks are identified in the Purcell factor spectrum around 5300 Hz and 12300 Hz. As a comparison, the Purcell factor predicted by the effective medium theory is plot as red dashed curve. (**b**,**c**) Near-field intensity distribution of the pressure wave at 5300 Hz (**b**) and 12300 Hz (**c**), respectively. It can be seen that at 12300 Hz the wave is mostly localized inside the central cavity, while at 5300 Hz, the wave is mainly distributed inside the central cavity, the annular cavity, and the narrow slits between them.

The ratio of the integrated wave power measured in the far-field with and without the enclosure is defined as acoustic Purcell factor^6^. In this regard, we use *P*_1_ and *P*_0_ to denote the integrated wave power measured in the far-field with and without the enclosure, respectively. Then the ratio of *P*_1_/*P*_0_ is the acoustic Purcell factor. In Fig. 2(a), we plot the acoustic Purcell factor of the enclosure as a function of frequency. It clearly exhibits two peaks with one and three orders of magnitudes larger than 1 at frequencies of 5300 Hz (*λ* = 64 mm) and 12300 Hz (*λ* = 27 mm), respectively, indicating significant enhancement of the acoustic source radiation at these frequencies. It is worth noting that the wavelengths at these two frequencies are both large compared to any characteristic length in the double-layer enclosure. Therefore, the enhanced source emission occurs in the sub-wavelength regime. The respective pressure intensity field distributions in the near-field at these frequencies are plotted in Fig. 2(b,c), both of which show the field is highly concentrated in the central cavity, while at 5300 Hz, the field also spreads into the air cavity between two concentric annuli.

**Figure 3:**
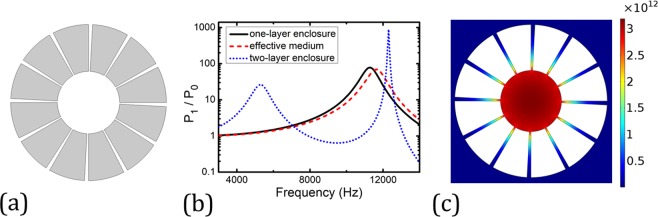
(**a**) Design of a one-layer enclosure, it is constructed by removing the outer ring of the two-layer enclosure and keeping the inner ring. (**b**) The acoustic Purcell factor (black solid curve) by a line source located at the center of the one-layer enclosure, and the Purcell factor predicted by the effective medium theory (red dashed curve). As a comparison, the Purcell factor for the two-layer enclosure is plot as the blue dotted curve. (**c**) Near-field intensity distribution of the pressure wave at 11300 Hz, where the wave energy is mostly localized inside the central cavity.

If we remove the outer annulus and keep the inner one, the enclosure becomes a single layer device as illustrated in Fig. 3(a). Similar behavior of the enhancement in the source emission rate still exists, but only one peak with a magnitude around 100 at frequency 11300 Hz (*λ* = 30 mm) is observed in the acoustic Purcell factor spectrum as shown in the black solid curve in Fig. 3(b). This peak frequency is close to that of the second peak discussed earlier for the double-layer case and the corresponding wavelength is still large compared to any characteristic size of the enclosure, such as the diameter (10 mm) and the width of the slits. The field distribution at this frequency is plotted in Fig. 3(c), whose pattern resembles that of the inner part shown in Fig. 2(c), implying the enhancement is mainly attributed to the inner annulus. For comparison, the acoustic Purcell factor for the double-layer case is also plotted in Fig. 3(b) in blue dotted curve, which exhibits a higher and sharper peak around the original resonance for the single layer case and a new peak at a lower frequency.

**Figure 4:**
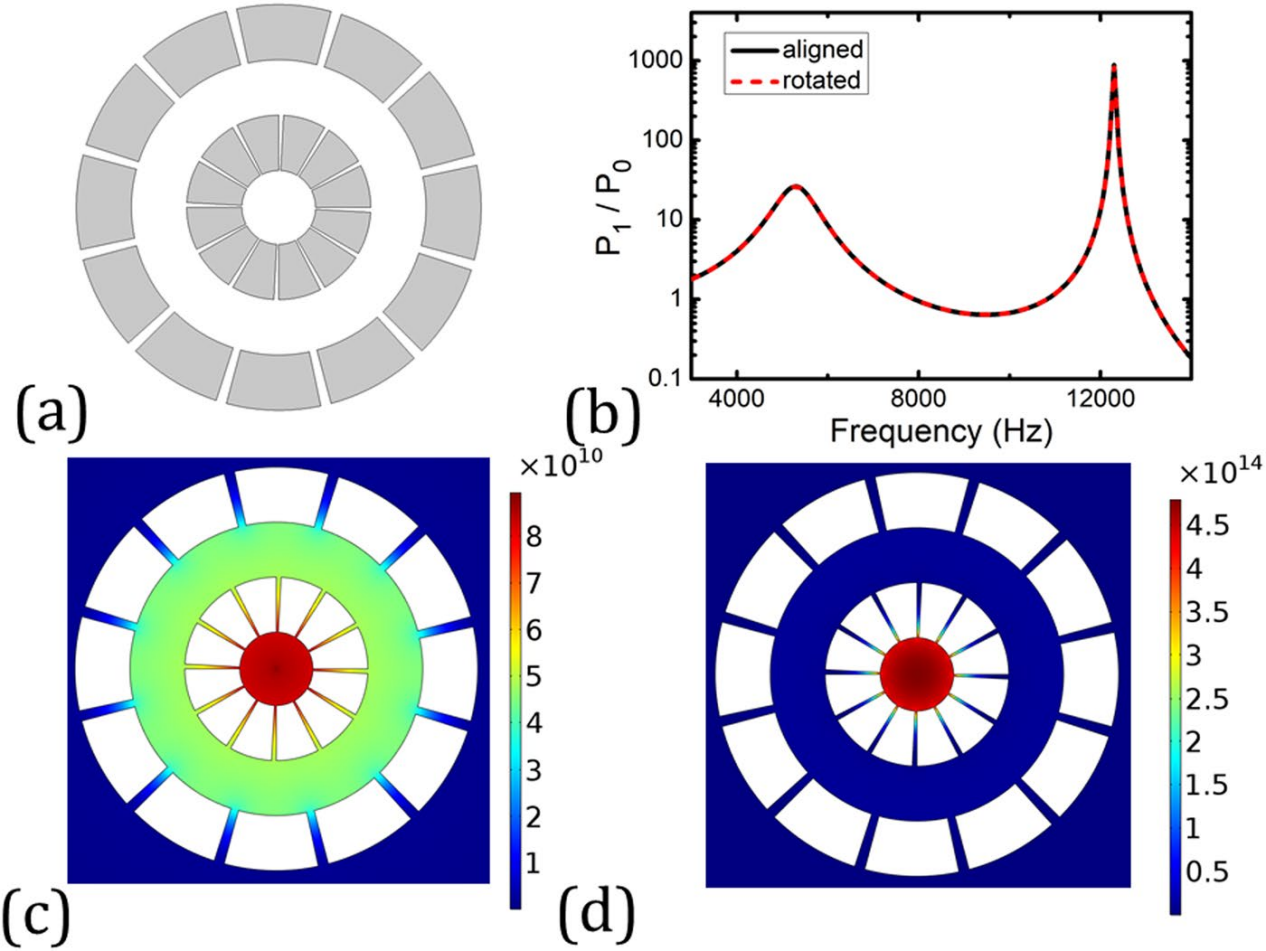
(**a**) Design of a new two-layer enclosure. The structure is the same as that shown in Fig. 1(a) except that the second layer is rotated along the azimuthal direction by 15 degrees with respect to the first layer. (**b**) The same radiation power spectrum is obtained for both structures shown in Figs 1(a) and 4(a). (**c**,**d**) Near-field wave intensity at 5300 Hz (**c**) and 12300 Hz (**d**), respectively.

We further rotate the outer annulus by 15° such that the slits in the outer annulus no longer align with those in the inner annulus as illustrated schematically in Fig. 4(a). Its corresponding acoustic Purcell factor is plotted in Fig. 4(b) in red dashed curve, which overlaps with the results of the aligned case represented by the black solid curve. The field distributions at the peak frequencies are plotted in Fig. 4(c,d), which exhibit the same behavior as shown in Fig. 2(c,d).

### Effective medium model

As per discussed earlier, the wavelengths at which the acoustic Purcell factor reaches its peaks are large compared to any characteristic size of the enclosure suggest the enclosure may be described by an effective medium, which is further affirmed by the results shown in Fig. 4 as the relative location of the slits does not affect the acoustic Purcell factor. In what follows, we aim at deriving a proper effective medium model to characterize the wave propagation behavior in the enclosure. Since the slits in each annulus are very thin, only the fundamental mode survives. By generalizing a coupled-mode theory developed for a planar slab with multiple cut-through slits to the circular case^5–14^, we can obtain the following anisotropic effective medium expressions for each annulus:

1 $$B=\frac{{B}_{air}}{f},\,{\rho }_{r}=\frac{{\rho }_{air}}{f},\,{\rho }_{\theta }=\infty .$$

Here, *B* is the effective bulk modulus, *ρ*_*r*_ and *ρ*_*θ*_ are the radial and angular components of the effective mass density, and *f* = 1/10 denotes the filling ratio of the slit. We would like to point out Eq. (1) is derived under the assumption that no wave is penetrated into the steel component. This assumption is valid because of the high contrasts in the mass density, bulk modulus and impedance between air and steel. To verify the effective medium model, we replace the two concentric annuli with their effective media whose parameters are given by Eq. (1) and repeat the simulation of acoustic Purcell factor. The results are plotted in Figs 2(a) and 3(b) in red dashed curves. Despite small discrepancies, generally good agreements between the results of the real structures and their effective media are manifested, validating the effective medium model. The small discrepancies may come from the modified effective thickness of the steel annuli^15^. When studying the eigenfrequencies of the steel annuli, we observe that there is nonnegligible pressure field leakage from the annuli’ slits into the background medium, which means that a slightly thicker effective medium may provide a more accurate description of the annuli structure. This may be the reason for the slight blue-shift of the Purcell factor predicted by the effective medium.

### Viscothermal effect

Since the slits’ width is small compared with the wavelengths at resonance peaks in the acoustic Purcell factor, the viscothermal effect for air inside the slits may become non-negligible. When considering the viscothermal losses in the slits, we adopt the expressions given by Michael R. Stinson^16^, i.e.,

2 $${\rho }_{air,v-th}=\frac{{\rho }_{air}}{1-tanh(\sqrt{-i}{{\lambda }}_{{s}})/(\sqrt{-i}{{\lambda }}_{{s}})}$$

3 $${B}_{air,v-th}=\frac{{B}_{air}}{1+({\gamma }-1)tanh(\sqrt{N}\sqrt{-i}{{\lambda }}_{{s}})/(\sqrt{N}\sqrt{-i}{{\lambda }}_{{s}})}$$

where *ρ*_*air*,*v*−*th*_ (*B*_*air*,*v*−*th*_) and *ρ*_*air*_(*B*_*air*_) are the mass density (bulk modulus) of air with and without viscothermal effect, respectively. In Eqs (2) and (3), $\sqrt{-i}=\frac{\sqrt{2}}{2}(1-i)$, *N* = 0.71 and *γ* = 1.4 are the Prandtl number and heat capacity ratio of air, respectively. ${{\lambda }}_{{s}}\,=b\sqrt{\omega /\nu }$, where *b* is the slit’s width, *ω* is the angular frequency, and *ν* = *μ*/*ρ*_*air*_ is a constant proportional to the dynamic viscosity *μ* = 18.5 ×1 0^-6^ Pa · s. It is evident from Eqs (2) and (3) that both mass density *ρ*_*air*,*v*−*th*_ and bulk modulus *B*_*air*,*v*−*th*_ take complex values when the viscothermal losses are included.

**Figure 5:**
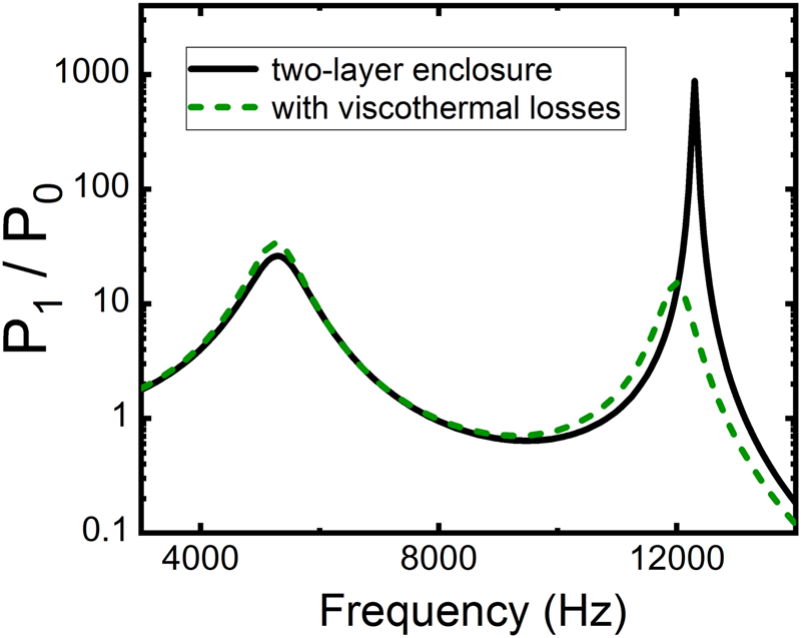
The acoustic Purcell factor by a line source located at the center of the enclosure, which has the same structure as that shown in Fig. 1(a). Black solid line and dark green dashed line represent the results calculated without and with viscothermal effect, respectively.

Assuming that air in the slit has a mass density and bulk modulus specified by Eqs (2) and (3), we have calculated the acoustic Purcell factor by a line source located at the center of the enclosure, and the results are shown in Fig. 5. We can observe that when viscothermal losses are included, there still exist two resonance peaks in the acoustic Purcell factor, as shown in dark green curve. The positions of the peaks, i.e., 5300 Hz and 12000 Hz, are close to those without considering viscothermal losses. The height of the first peak is almost not affected by the losses, but the second one is substantially reduced. This is reasonable. Usually high frequency sounds dissipate a larger amount than low frequency sounds, simply because they have a shorter wavelength.

We also notice that the effective medium expressions given in Eq. (1) can be easily modified to accommodate the situations where the viscothermal effects are included. In such situations, we need to replace *B*_*air*_ and *ρ*_*air*_ by *B*_*air*,*v*−*th*_ and *ρ*_*air*,*v*−*th*_, respectively and the resulted effective parameters (*B*, *ρ*_*r*_ and *ρ*_*θ*_) are complex numbers.

## Discussion

**Figure 6:**
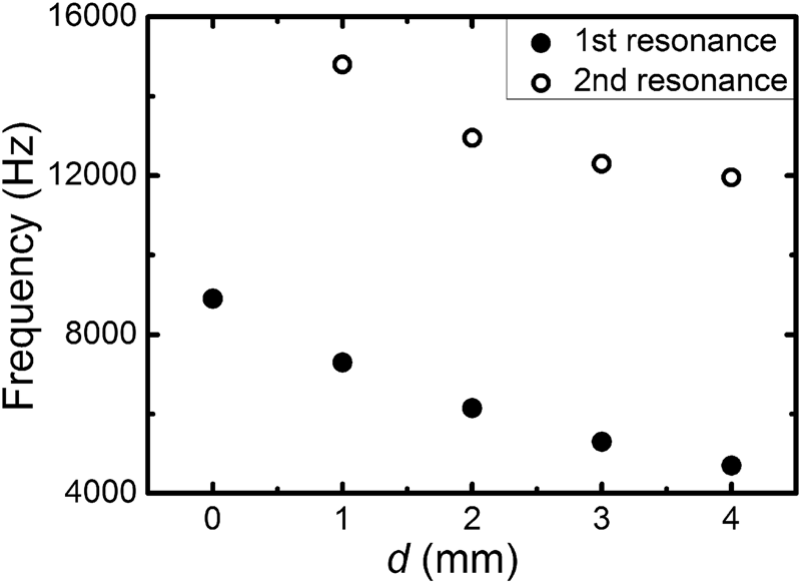
Dependence of the 1^st^ (lowest) and 2^nd^ (second lowest) resonance frequencies on the separation *d* between the two annuli. The inner radius is fixed at 2 mm, and the thickness of both annuli is kept at 3 mm. The slits in the outer annulus always align with those in the inner annulus. Please note that *d* = 0 mm means the two layers merge into one single layer with a total thickness of 6 mm.

All of the results presented so far indicate the enhanced acoustic source emission by the double-layer enclosure strongly depends on its geometry. Figure 6 demonstrates the dependence of the frequencies of the first and second resonance peaks in the acoustic Purcell factor on the separation between two annuli while the inner one is fixed and thickness of both annuli is kept at 3 mm. Here the slits in the outer annulus always align with those in the inner annulus. When the separation distance *d* vanishes, the two annuli merge into a single one with a thickness of 6 mm and there only exists one peak. It is observed from Fig. 6 that the frequencies for both resonance peaks decrease as the separation between the rings increases. This behavior can be qualitatively understood from a simple physical picture. We can see from the pressure intensity field presented in Fig. 2(b,c) that at the resonance frequencies most of the wave energy is distributed inside the central cavity and the in-between cavity. When the separation distance *d* increases, the space of the in-between cavity also increases, offering more space available for the cavity modes, and therefore resulting in more extended cavity modes. From the reciprocal relationship between the spatial coordinate (as measured by the wavelength *λ*) and frequency, the resonance frequency will be lowered when a more spatially extended mode is obtained^8^. Figure 6 also shows the 1^st^ resonance frequency changes more rapidly than the 2^nd^ one as *d* increases, because the 1st resonance depends heavily on the in-between cavity, which is manifested by the field pattern shown in Fig. 2(b). This observation provides a design principle to achieve enhanced source emission at low frequencies: by introducing proper in-between cavities. Such principle is fundamentally different from the strategy proposed previously in ref.^6^, where the low frequency feature is mainly contributed by the elongated acoustic path in the labyrinthine structure.

**Figure 7:**
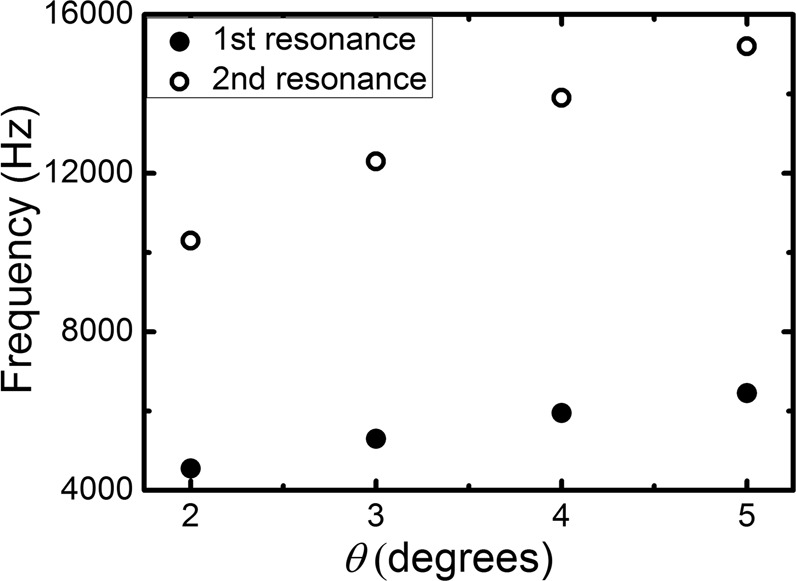
Dependence of the 1^st^ (lowest) and 2^nd^ (second lowest) resonance frequencies on the angle *θ* of the slit’s width. All other geometrical parameters are the same as those specified in Fig. 1(a).

We further alter the size of the slits while keeping the thicknesses and radii of the annuli unchanged and studying the influences on the resonant frequencies. The results are plotted in Fig.7, which shows the wider the slits, the higher the frequencies for both the first and second resonances. This behavior can be interpreted from a simple perspective: if the slits become larger and larger such that the filling ratio approaches 1, the acoustic Purcell effect vanishes. That means the low frequency peak in the acoustic Purcell factor disappears eventually. Thus, as the slits become wider, the peaks shift to higher frequency. A more rigorous analysis based on mode expansion for single planar slab perforated with periodic subwavelength holes was discussed in ref.^15^, which reveals the resonant frequency of the slab increases as the filling ratio of the holes increases because of the interaction between the holes through the evanescent waves. Indeed, similar mechanism also exists in our proposed structure.

Although we focus on the enhanced emission of the acoustic monopole, the enhancement also occurs for multipoles, which is related to an interesting type of resonances, the so-called degenerate Mie resonance, where the Mie resonant frequencies of different orders overlap. The degenerate Mie resonance is enabled by the super-anisotropic feature of the effective mass density^5^. The solution to the acoustic wave equation in an effective medium with parameters given by Eq. (1) can be written as:

4 $$p \sim \{\begin{array}{c}{J}_{v}(\omega r/{c}_{r})\\ {H}_{v}^{(1)}(\omega r/{c}_{r})\end{array}\}{e}^{\pm im\theta }{e}^{-i\omega t};v=m\sqrt{\frac{{\rho }_{r}}{{\rho }_{\theta }}}\approx 0,$$

where ${J}_{v}(\,\cdot \,)$ and ${H}_{v}^{(1)}(\,\cdot \,)$ are the Bessel function and Hankel function of the first kind of order *v*, respectively, and *m* is an integer. An ${e}^{-i\omega t}$ time dependence is assumed for all variables, where $\omega$ is the angular frequency and ${c}_{r}$ represents the velocity along the radial direction. It is not difficult to find that only the zero-*th* order Bessel function and Hankel function of the first kind survives in the solution, regardless of the value of *m*, which is attributed to the extremely large angular component of the mass density that forces *v* to be zero. This feature differs drastically from those common cases with isotropic enclosure and results in the degeneracy of Mie resonances with different orders. The degenerate Mie resonances help enhancing the density of states for both the monopole and multipole sources^5,14^.

## Summary

In this work, we propose a simple ‘LEGO’-type design of acoustic metamaterial that comprises a dual grating structure to enhance the acoustic source emission rate at low frequencies. Different from previous work that based on either Fabry-Perot resonance or the reduced effective acoustic speed by elongated acoustic path, our metamaterial attributes its functionality to the coupling between the cavity modes and the resonances of the dual grating. An anisotropic effective medium model is developed to characterize the enhancement, and the dependence of the enhancement on various geometric parameters is explicitly studied. Since our structure is simple to fabricate, we believe it will benefit the communities that desires high acoustic source emission rate.

## Methods

### Numerical simulations

All the numerical simulations presented in this article are performed using COMSOL Multiphysics, a commercial package based on the finite-element method. The pressure field patterns presented in Figs 1–4 are calculated using the frequency domain study in the pressure acoustics module, where a cylindrical wave radiation boundary condition is imposed on the outer boundary in the far-field so that there is no reflected wave from the domain boundary. In the simulations, sound hard boundary conditions are imposed on the boundaries of the steel annuli.
